# Psychometric properties of the Problematic Online Gaming Questionnaire (POGQ) in a Moroccan sample of university students

**DOI:** 10.1186/s40359-023-01437-3

**Published:** 2023-11-16

**Authors:** Samira Abbouyi, Samira Bouazza, Jaouad El Hilaly, Mohammed El Amine Ragala, Karima El Rhazi, Btissame Zarrouq

**Affiliations:** 1 Laboratory of Epidemiology and Research in Health Sciences, Faculty of Medicine, Pharmacy and Dental Medicine, Sidi Mohamed Ben Abdellah University, KM 2.200 Route Sidi Harazem, 30070 Fez, Morocco; 2 Laboratory of Pedagogical and Didactic Engineering of Sciences and Mathematics, Regional Center of Education and Training (CRMEF) of Fez. Rue Koweit, P.B 49 Agdal, 30050, Fez, Morocco; 3 Teachers Training College (Ecole Normale Superieure), Department of Biology and Geology, Sidi Mohamed Ben Abdellah University, P. B 5206 Bensouda, 30030, Fez, Morocco

**Keywords:** Problematic online gaming questionnaire (POGQ), Psychometric properties, Validity, Reliability, Moroccan context, Arabic language

## Abstract

**Background:**

The Problematic Online Gaming Questionnaire (POGQ) instrument consists of 18 items with a six-factor structure. This questionnaire is widely utilized to measure the degree of problematic online gaming, but the scale has not, up to date, been validated in Arabic language. This study aimed to assess POGQ scale validity and reliability in Moroccan context.

**Methods:**

The research was conducted from April to June 2023 using an online questionnaire. The selected sample involved Moroccan university students. Data were analyzed in two successive phases. First, exploratory factor analysis (EFA) was used to assess the factor structure in the first sample (n1 = 143). Then, this structure was confirmed in the second sample (n2 = 313) using confirmatory factor analysis (CFA).

**Results:**

The EFA and CFA results demonstrated that the POGQ has a six-factor structure explained 72% of the total variance. The results of this analysis provided an optimal fit to the data confirming a good performance of the measurement model (χ² = 243.6; CFI = 0.981; TLI = 0.976; RMSEA = 0.048; NFI = 0.964; IFI = 0. 981; SRMR = 0.022). The instrument showed sufficient reliability and convergent validity demonstrated by acceptable values of composite reliability (CR = 0.68–0.90), and average variance extracted (AVE = 0.50–0.75), respectively. Finally, the Arabic version of POGQ was found to have a high test–retest reliability.

**Conclusions:**

The Arabic version of POGQ revealed adequate psychometric properties. As a result, the instrument might be used to measure the degree of problematic online gaming. The use of the POGQ is expected to further promote research on online game dependence treatment and prevention.

## Background

Digital technologies have become extensively utilized worldwide [1]. With the increasing use of these technologies, an increasing minority of individuals have been found to exhibit problematic behaviors associated with them [2]. In fact, it seems that each new digital technology and platform generates a small group of individuals who experience difficulties with the technology, which some researchers describe as addictions [3, 4]. The initial paper on internet addiction was published by Griffiths [5], who, like Young [6], used it to describe individuals who spent excessive amounts of time online, neglecting other aspects of their lives. However, more than two decades later, the term has evolved into an umbrella term primarily used to describe addictions on the Internet rather than to the Internet. Now, terms such as “problematic,“ “addictive,“ “pathological,“ and “compulsive” are commonly used in conjunction with Internet and smartphone use in general [6–13], as well as specific online activities such as online shopping [14], online gambling [15, 16], online pornography [17], and online gaming [2].

The World Health Organization (WHO) formally recognized gaming disorder (GD) as a disorder due to addictive behaviors in May 2019, incorporating it into the eleventh revision of the International Classification of Diseases (ICD-11) under the category “Disorders due to substance use or addictive behaviors” [18]. This decision, rooted in accumulated evidence, was the outcome of extensive discussions among global experts [19, 20]. According to the ICD-11 definition of GD, a diagnosis requires meeting three clinical manifestation criteria and one functional impairment criterion [18]. Furthermore, these behavioral patterns and impairments must persist for at least 12 months, except in cases where severe symptoms are present.

Prior to its inclusion in the ICD-11, the American Psychiatric Association outlined preliminary diagnostic criteria for internet gaming disorder (IGD) in the fifth edition of the Diagnostic and Statistical Manual of Mental Disorders (DSM-5) in 2013 [15]. According to the guidelines outlined in the DSM-5, clinical diagnosis of IGD requires the fulfillment of at least five out of nine criteria within a 12-month period. These nine criteria encompass: [1] developing preoccupation with Internet games, [2] experiencing withdrawal symptoms when the Internet game is unavailable, [3] developing tolerance with increased time spent on Internet games, [4] inability to control participation in Internet games, [5] loss of interest in previous hobbies and entertainment due to Internet games, [6] persistent engagement in Internet games despite being aware of the negative impacts (i.e., psychosocial problems), [7] lying to family members, therapists, and others about the amount of time spent on Internet gaming, [8] using Internet games as a means of escape from negative moods or feelings, and [9] losing significant relationships or other opportunities (e.g., job/career or education) due to excessive Internet gaming.

While the DSM-5 and ICD-11 assert a commonality between disordered gaming, substance use, and gambling disorders [21], the diagnostic criteria for GD continue to be a topic of contention. A Delphi expert consensus method was applied to assess the diagnostic validity, clinical utility, and prognostic value of DSM-5 and ICD-11 criteria for GD [22]. Involving 29 international experts, the three-round survey revealed consensus on certain DSM-5 Internet Gaming Disorder criteria, emphasizing their validity, clinical utility, and prognostic value. However, some criteria, such as tolerance and deception, were considered less relevant. Notably, specific DSM-5 criteria, like escapism/mood regulation and tolerance, were viewed as unable to distinguish between problematic and non-problematic gaming, risking the pathologization of the latter. In contrast, ICD-11 diagnostic guidelines for GD, with the exception of a criterion related to diminished non-gaming interests, were generally regarded as possessing high diagnostic validity, clinical utility, and prognostic value [22].

Research has shown that IGD is linked to specific personality traits such as psychopathology [23–25], neuroticism [23, 26], poor psychological wellbeing [24, 27, 28], impulsivity [24, 29, 30], lower academic performance [31, 32], lower social connectedness [28, 33], poor interpersonal relationships [15, 30], and poor sleep quality [31, 34]. Studies have also examined the relationship between IGD and mental health, especially psychological distress [25, 35]. Empirical studies have demonstrated that IGD has been associated with depression [36–39], anxiety [38, 39], and social anxiety [40–42]. In light of these findings, it can be concluded that IGD exhibits a range etiological spectrum. Moreover, there is a forecast that IGD will emerge as a noteworthy health concern for a minority of individuals in the foreseeable future. Studies have further shown that adolescents and emerging adults, especially males, are identified as a demographic at risk for developing IGD [25, 43].

The prevalence of Internet Gaming Disorder (IGD) globally ranges widely, from 0.7 to 27.5%, influenced by factors such as study design, measurement methods, and demographic factors, with younger individuals exhibiting higher rates than older age groups and males reporting a higher prevalence than females [44]. A comprehensive review of 160 studies, utilizing 35 different diagnostic methods, demonstrated a prevalence range of 0.21–57.5%, influenced by geography, gender, and age groups [45]. Specific populations have been investigated, revealing distinct patterns. A meta-analysis of 16 worldwide studies published before 2017 found a pooled prevalence of 4.6% among adolescents, varying from 0.6 to 19.9%, with higher rates observed among males [46]. Research on children indicates a 1.5% addiction prevalence among those aged 13 to 16 years [47]. Among medical students, the pooled prevalence of IGD is 6.2% [48]. In nine African countries, 30% of gamers were addicted, 30% were problematic, 8% were engaged, and 32% were non-problematic, while Morocco showed figures of 27.53% addicted, 27.14% problematic, 8.57% engaged, and 36.76% non-problematic [49].

To obtain reliable prevalence data, it is crucial to utilize psychometrically validated measurement tools [50]. Unfortunately, there is a lack of such tools, and many questionnaires have been adapted from other measures without undergoing rigorous reliability and validity testing. These include tools based on Internet addiction (e.g., Internet Addiction Test) [6], pathological gambling (using DSM-IV criteria), or behavioral addictions [51, 52]. Another challenge is that many existing tools primarily focus on Massively Multiplayer Online Role Playing Games (MMORPGs) [53, 54].

The Problematic Online Gaming Questionnaire (POGQ), which consists of 18 items with a six-factor structure is widely utilized to measure the degree of problematic online gaming. By employing this scale, it becomes possible to comprehend the individual and social issues caused by online games across six factors: preoccupation, overuse, immersion, social isolation, interpersonal conflict, and withdrawal. The POGQ serves as a valuable measurement tool and aids in the investigation of clinical problems such as social isolation, interpersonal conflict, and withdrawal. Furthermore, the POGQ has been translated into Japanese [55], Finnish [56], Italian [57], Hungarian [58], shortened [59], and applied in various regions and cultures, expanding its applicability. In the Arab community such as Morocco, research on problematic online gaming is still in its early stages, and this topic remains largely unexplored. One reason for this is the lack of a comprehensive set of indicators for assessing symptoms related to problematic online gaming. Hence, it is necessary to translate the POGQ and validate an Arabic version to tackle the issues associated with online gaming. Consequently, this study aimed to adapt an Arabic version of POGQ scale, and explore its validity and reliability properties in Moroccan context.

## Methods

### Study design and participants

Various recommendations have been proposed for determining sample size based on participant-to-item ratios. Cattelle (1978) and Hogarty et al. (2005) propose a minimum ratio of 3 [60, 61], while Everitt (1975) and Henson and Roberts (2006) argue for a ratio exceeding 10 [62, 63]. The commonly used guideline suggests a participant-to-item ratio of 10:1 [64, 65], with an ideal ratio of 20:1 [61]. Although Costello and Osborne (2005) advocate for larger sample sizes for more accurate results [66], there is no consensus on the required size for performing EFA [61]. Hair et al. (2014) recommend a sample size larger than 100 and at least five times as many observations as variables [67]. However, these guidelines face criticism for neglecting item communality, overestimation of factors, and loading sizes [68, 69]. Instead, it is recommended that researchers recruit as a large sample as practical because sample adequacy cannot be determined until after the data have been analysed [63]. Worthington and Whittaker (2006) propose that if item communalities are ≥ 0.50 or there are 10:1 items per factor with loadings around 0.40, a sample size of 150–200 may be sufficient. For higher communalities (≥ 0.60) or a minimum of 4:1 items per factor with factor loadings above 0.60, then smaller samples may be adequate [70]. Based on these recommendations, the sample size in this cross-sectional study, conducted between April and June 2023 among Moroccan university students aged 18 years or above, included 456 subjects in the final analysis, meeting the suggested sample size criteria. Additionally, gathering over 100 people was deemed necessary for the analysis criteria of test–retest reliability [71].

### Measures

Problematic online gaming was assessed using the Problematic Online Gaming Questionnaire (POGQ). The POGQ instrument was developed by Demetrovics et al. (2012). It comprises 18 items using a 5-point Likert scale (from 1 = never to 5 = always), with higher scores reflecting a greater tendency toward online problematic gaming. This scale measures six dimensions of problematic use: Preoccupation (two items) refers to daydreaming and obsessive thinking about the online gaming; immersion (four items) refers to losing track of time and dealing excessively with games; withdrawal (four items), refers to experiencing withdrawal symptoms when unable to play; overuse (three items) refers to elongated gaming time and incapacity to control gaming limits; interpersonal conflicts (two items) refer to conflict with one’s environment due to excessive play; and the last dimension, social isolation (three items) refers to preference of gaming over social relationships and activities.

### Translation process

The POGQ scale was translated back and forth between English and Arabic. First, an Arabic version of the translation was made by two independent translators. Then, The Arabic version was then translated back into English by two separate translators without consulting the original English text. Finally, the differences between the two English versions of the POGQ (i.e., the original and back-translated versions) were discussed, and only minor discrepancies were found. These discrepancies were discussed until a consensus was reached. After it was estimated as satisfying, the committee decided on the final Arabic version. 20 university students pre-tested this POGQ version in order to evaluate whether that it was clear. No item was found to be difficult to understand. Therefore, no revision was made after the pilot.

### Data collection

In this study, data were collected with an online questionnaire; filling it out took about 10 minutes. To assemble our sample, we identified and recruited ten individuals, aptly named ‘seeds’. These ‘seeds’ were not randomly selected; rather, they were specifically chosen to ensure a diverse representation in terms of age, gender, and academic level to our target population. Each ‘seed’ played a pivotal role by acting as a disseminator of the questionnaire link within the student community. These individuals employed a multi-faceted approach, leveraging platforms like WhatsApp groups, Facebook groups, and other social media channels. This strategic dissemination aimed to reach different regions of Morocco and various spheres of the student population, fostering participation from a broad spectrum of potential respondents with diverse geographic backgrounds.

The inclusion criteria for this study involved game users aged 18 years or above, enrolled in a Moroccan university. Individuals not meeting these criteria were excluded from participation.

All participants volunteered to take part in the study, and no financial incentives or compensation were provided for their involvement. Prior to their participation, we obtained electronic informed consent from each participant, ensuring their agreement to continue and participate in the study. Following this, we asked for some sociodemographic data, such as age, gender, residence, and the year of study in the university. Moreover, they were asked to report about the Internet usage time per day. This section was followed by the Arabic version of the Problematic Online Gaming Questionnaire (POGQ). All these items were answered via a Google form between April 23, 2023, and June 18, 2023. To verify the test–retest reliability of the Arabic version of POGQ, participants were selected from the initial sample based on their voluntary participation and willingness to provide responses for the follow-up assessment. We ensured a diverse representation by including individuals from different demographic backgrounds, including age, gender, and academic levels. Those who had completed the initial questionnaire and expressed their consent for the retest were invited to participate in the follow-up assessment approximately two weeks after their initial response. This process aimed to capture a reliable and varied subset of the original participants for assessing the test-retest reliability of the Arabic version of POGQ. This study has been approved by the hospital-university ethics committee of Sidi Mohamed Ben Abdellah University (N°16/22).

### Data analysis

The statistical analyses were conducted using IBM SPSS statistics software version 25 software and JASP version 16 software. Correlations were evaluated by the Pearson coefficient r. Exploratory Factor Analysis (EFA) was performed to explore the factor structure of the Arabic version of the POGQ on the first sample (n1 = 143), which was randomly selected from the initial participant pool. Principal axis factoring (PAF) with a promax rotation was used as an extraction method. To determain the number of factors to retain during the Exploratory Factor Analysis (EFA), we employed the Kaiser-Guttman criterion, retaining factors with eigenvalues surpassing 1. Additionally, elements with a factor loading exceeding 0.40 were retained, while others were excluded [72–74]. The goodness of fit was assessed by the root-meansquare error of approximation (RMSEA), Tucker Lewis Fit Index (TLI), and chi square (χ2). Moreover, a confirmatory factor analysis was carried out to confirm the factor structure of the Arabic version of the POGQ on the second sample (n2 = 313). The goodness of fit was evaluated using RMSEA and its 90% confidence interval (90% CI), p value smaller than 0.05 for test of chi-square (χ2), standardized root-meansquare residual (SRMR), comparative fit index (CFI), and Tucker Lewis Fit Index (TLI). The reliability was assessed by Cronbach’s alpha coefficient. Furthermore, the internal consistency and convergent validity were estimated by computing Composite Reliability (CR) and Average Variance Extracted (AVE) correspondently. The test-retest reliability was evaluated using the Intraclass Correlation Coefficient (ICC). This coefficient ranges from 0 to 1, with values below 0.5 indicating poor reliability, 0.5 to 0.75 denoting moderate reliability, 0.75 to 0.9 indicating good reliability, and any value surpassing 0.9 signifying excellent reliability [75].

## Results

### Demographic data

This study involved two samples of Moroccan university students. The first (n1 = 143) was analyzed by EFA, while the second (n2 = 313) was tested by CFA. Within the first sample, more than half of the participants were female (55.9%), whereas in the second sample, the percentage of female participants was 46.6%. Regarding their living arrangements, a significant proportion of participants in both samples resided with their parents (62.9% in the first sample and 65.8% in the second sample), and a considerable majority of the participants were single (79.7% in the first sample and 89.1% in the second) (Table 1).

**Table 1:** Sociodemographic characteristics of participants (N = 456)

Participants characteristics	n1 = 143	n2 = 313
	**n (%)**	**n (%)**
**Gender**		
Female	80 (55.9)	146 (46.6)
Male	63 (41.1)	167 (53.4)
**Age**		
18–20	28 (19.6)	76 (24.3)
21–23	31 (21.7)	84 (26.8)
24–26	29 (20.3)	72 (23.0)
More than 26	55 (38.5)	81 (25.9)
**Marital status**		
Single	114 (79.7)	279 (89.1)
Married	28 (19.6)	33 (10.5)
Divorced	1 (0.7)	1 (0.3)
**University Level**		
Freshman or Sophomore	33 (23.1)	84 (26.8)
junior	66 (46.2)	143 (45.7)
Master	37 (25.9)	67 (21.4)
PhD student	7 (4.9)	19 (6.1)
**Study specialty**		
Scientific	60 (42.0)	144 (46.0)
Medical	11 (7.7)	21 (6.7)
Literary	28 (19.6)	59 (18.8)
Technical	9 (6.3)	25 (8.0)
Economics	26 (18.2)	38 (12.1)
Law	9 (6.3)	26 (8.3)
**Living arrangements**		
Alone	24 (16.8)	29 (9.3)
With friends	27 (18.9)	66 (21.1)
With parents	90 (62.9)	206 (65.8)
With other family members	2 (1.4)	12 (3.8)
**Internet subscription**		
Yes	102 (71.3)	210 (67.1)
No	41 (28.7)	103 (32.9)
**Time spent on the Internet (hours)**		
< 1	3 (2.1)	5 (1.6)
1	5 (3.5)	16 (5.1)
2–3	46 (32.2)	120 (38.3)
4–5	50 (35.0)	94 (30.0)
6 or more	39 (27.3)	78 (24.9)

### Exploratory factor analysis

First, before conducting EFA, the appropriateness of the data was assessed using the Kaiser-Meyer-Olkin (KMO) coefficient, which yielded values exceeding 0.91 for all individual items. Additionally, Bartlett’s Sphericity Test (χ2 = 6564.73, df = 153, p < 0.001) indicated that the inter-item correlations were substantial enough to justify conducting EFA [76]. A loading threshold of at least 0.40 was initially applied.

**Fig. 1 CFA measurement model:**
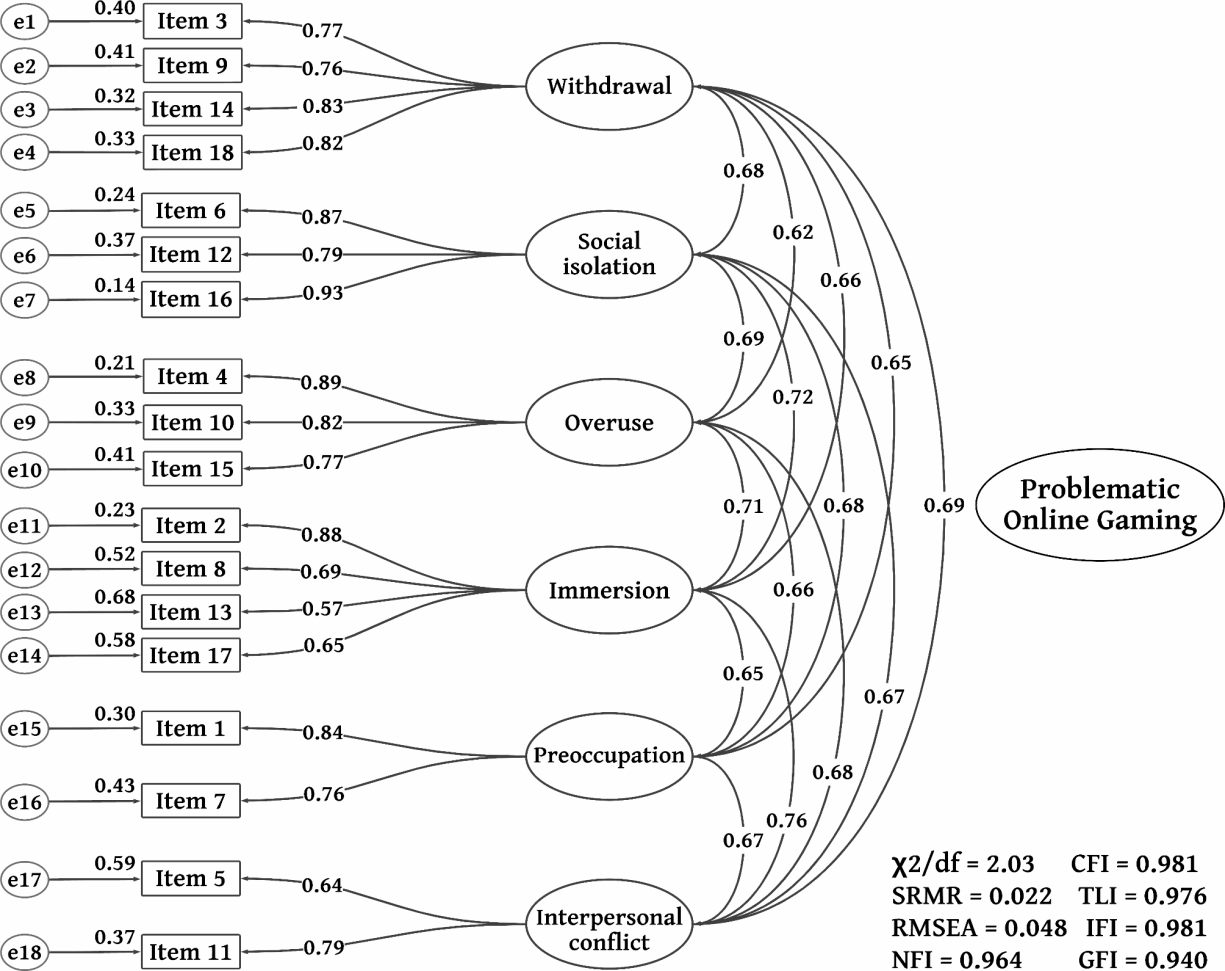
χ2 Chi-square test; df Degrees of freedom; RMSEA Root Mean Square Error of Approximation; CFI Comparative Fit Index; SRMR Standardized Root Mean Square Residual; TLI Tucker-Lewis Index; IFI Incremental Fit Index; NFI Normed Fit Index; GFI Goodness of Fit Index

Subsequently, EFA was performed on the data from the initial sample (n1 = 143) using principal axis factoring (PAF) as extraction method, with promax rotation. Extracted factors were determined by the Kaiser-Guttman criterion, retaining those with eigenvalues surpassing 1 and factor loadings above 0.40 [72–74]. No items were eliminated, and six factors resembling the original POGQ version were extracted, explaining 72% of the variance. These six constructs were labeled as Withdrawal (4 items), Social isolation (3 items), Overuse (3 items), Immersion (4 items), Preoccupation (2 items), and Interpersonal conflict (2 items). Factor loadings values were ranged between 0.57 and 0.93 (Table 2; Fig. 1). A loading threshold of at least 0.40 was initially applied. The goodness-of-fit indicators exhibited very favorable results (χ2/df = 2.58, RMSEA = 0.05, TLI = 0.96) [77].

### Internal consistency

To evaluate the reliability of the Arabic version of POGQ, we assessed its internal consistency by calculating the Cronbach’s α coefficient for each construct (as shown in Table 2). The construct labeled ‘interpersonal conflict’ exhibited the lowest alpha value of 0.84, while the alphas for the remaining subscales ranged from 0.85 to 0.93. These findings confirm a very strong level of internal consistency across the different constructs. Typically, alpha values should be at least 0.70 to be considered as having good internal consistency, and preferably higher than 0.80 for even stronger consistency. In this case, all of the constructs meet or exceed these criteria, indicating that the Arabic version of POGQ demonstrates excellent reliability (Table 2).

**Table 2:** Factor loadings for the CFA of the Arabic version of POGQ

Items	Factors ^a^					
	WI	SI	OV	IM	PR	IC
POGQ14	0.83					
POGQ 18	0.82					
POGQ 9	0.76					
POGQ 3	0.77					
POGQ 16		0.93				
POGQ 6		0.87				
POGQ 12		0.79				
POGQ 4			0.89			
POGQ 10			0.82			
POGQ 15			0.77			
POGQ 2				0.88		
POGQ 8				0.69		
POGQ 17				0.65		
POGQ 13				0.57		
POGQ 1					0.84	
POGQ 7					0.76	
POGQ 11						0.79
POGQ 5						0.64
Mean (SD)	1.58 (0.76)	1.58 (0.86)	1.59 (0.86)	1.87 (0.85)	1.65 (0.83)	1.62 (0.89)
Cronbach’s alpha	0.89	0.93	0.92	0.86	0.85	0.84
Variance explained (Total = 72%)	15%	14%	13%	14%	08%	08%

^a^Abbreviations for the original POGQ subscales: WI Withdrawal; SI Social isolation; OV Overuse; IM Immersion; PR Preoccupation; IC Interpersonal conflict

### Test–retest reliability

A total of 102 participants (41 male, 61 female) were included in the test-retest reliability analysis of the Arabic version of POGQ, which fulfilled the criteria for being excellent, with ICC (2, 1) = 0.973, p < 0.001, and 95% CI = 0.967–0.979.

### Confirmatory factor analysis

To test the original six-factor model of the POGQ we conducted a confirmatory factor analysis (CFA). The results of this analysis provided an optimal fit to the data (χ² = 243.6, p < 0.001, df = 120; RMSEA = 0.048 [0.039–0.056]) (Table 3; Fig. 1).

**Table 3:** Overall fit indices of the CFA model

Fit index	χ2/df	RMSEA	SRMR	GFI	NFI	CFI	TLI
Observed Value	2.03	0.048	0.022	0.940	0.964	0.981	0.976
Level of acceptance	< 3	< 0.05	< 0.08	> 0.90	> 0.90	> 0.90	> 0.90

χ2 Chi-square test; df Degrees of freedom; RMSEA Root Mean Square Error of Approximation; CFI Comparative Fit Index; SRMR Standardized Root Mean Square Residual; TLI Tucker-Lewis Index; NFI Normed Fit Index; GFI Goodness of fit Index

In the six-factor model, the factor loadings of the POGQ in both the EFA and CFA samples ranged from 0.57 to 0.93, further demonstrating that all the items effectively measured the problematic online gaming construct. Moreover, it underscores the scale’s robust psychometric properties and a solid factor structure. Furthermore, the reliability and convergent validity of the instrument were substantiated, with consistently high values for the Composite Reliability (CR) ranging from 0.68 to 0.90 and the Average Variance Extracted (AVE) ranging from 0.50 to 0.75, as detailed in Table 4. These findings affirm the entire process of factor analysis and demonstrate that the POGQ instrument fits the data quite well.

**Table 4:** Composite reliability, average variance extracted, and correlations between factors

Factors	CR	AVE	Correlation between factors^a^					
			WI	SI	OV	IM	PR	IC
Withdrawal	0.87	0.63	1	0.68	0.62	0.66	0.65	0.69
Social isolation	0.90	0.75		1	0.69	0.72	0.68	0.67
Overuse	0.87	0.68			1	0.71	0.66	0.68
Immersion	0.79	0.50				1	0.65	0.76
Preoccupation	0.78	0.64					1	0.67
Interpersonal conflict	0.68	0.52						1

CR Composite Reliability; AVE Average Variance Extracted

^a^Abbreviations for the original POGQ subscales: WI Withdrawal; SI Social isolation; OV Overuse; IM Immersion; PR Preoccupation; IC Interpersonal conflict

## Discussion

Using a sample of Moroccan university students, the present study sought to conduct a psychometric validation of the Arabic version of the POGQ in an attempt to create an instrument with sound psychometric properties that could be applied in the Arab cultural context to stimulate research on problematic online gaming. According to this aim, the POGQ was tested in a cross-sectional study using an online survey to recruit online gamers. This scale includes 18 items divided into six dimensions: preoccupation, overuse, immersion, social isolation, interpersonal conflict, and withdrawal. The POGQ was assessed in terms of validity and reliability from several levels. In regard to the structural validity of POGQ was evaluated using both exploratory factor analysis (EFA) and confirmatory factor analysis (CFA). The EFA and CFA results demonstrated that the POGQ has a six-factor structure explained 72% of the total variance and is more than sufficient, further supporting the six-dimensional factor structure of the POGQ found in previous studies [55–58].

Furthermore, the reliability of POGQ was evaluated with Cronbach α internal consistency reliability coefficient. In the social sciences, an internal consistency reliability coefficient of 0.70 and above is considered sufficient for such scales [78]. In the original study, the POGQ showed adequate reliability (α of 0.93), whereas Cronbach’s alphas ranged between 0.84 and 0.93 in subsequent studies [55–59]. It was 0.84 for the Hungarian version [58] and 0.91 for the Italian [57], Japanese [55] and the short version [59] and 0.87 for the Finnish version. Consistent with these reliability findings previously reported, the Arabic version reached similar results regarding the reliability of the POGQ (Cronbach’s alpha of 0.95).

The POGQ exhibited high internal consistency, with alpha coefficients exceeding 0.8, indicating strong reliability and high test-retest reliability, measured by the intraclass correlation coefficient (0.97), further supported the stability of the instrument over time. Convergent validity was evident through substantial Average Variance Extracted (AVE) values ranging from 0.50 to 0.75, indicating the POGQ’s ability to measure the intended construct consistently. These findings align with other studies validating the POGQ. For instance, the Italian version demonstrated robust convergent validity when correlated with the Problematic Internet Use Questionnaire Short Form (PIUQ-6; r ¼ 0.68, p < 0.001) and the Global Severity Index (GSI; r ¼ 0.51, p < 0.001) [57]. The Japanese version, in a similar vein, established convergent validity through associations with time spent on online gaming (r = 0.309, p < 0.001), the Game Addiction Scale for Adolescents (GAS7; r = 0.824, p < 0.001), and the EuroQol 5 Dimension 5-level (EQ-5D-5 L; r = 0.291, p < 0.001) [55]. The findings of the present study indicate that the Arabic version of POGQ is a valid and reliable scale can provide a valid and reliable measure of Problematic online gaming with excellent diagnostic accuracy that can be used in research and for diagnostic purposes among young adult male and female gamers. Overall, previous validity studies for the POGQ [55–59] were corroborated by the findings of this research.

The POGQ scale validated in this study assesses the problematic use of online games through six dimensions, namely preoccupation, immersion, withdrawal, overuse, interpersonal conflicts, and social isolation. These factors offer valuable insights into the dimensions of online gaming behavior that may align with the diagnostic criteria outlined in the 11th Revision of the (ICD-11) for gaming disorder. The factors of preoccupation and immersion reflect a cognitive preoccupation and intense involvement with online gaming, respectively. These align with the ICD-11 criterion of impaired control over gaming, emphasizing the persistent and escalating nature of gaming behavior that interferes with other aspects of life. Withdrawal symptoms, as indicated by the withdrawal factor, and overuse, characterized by elongated gaming time and a lack of control, correspond to the ICD-11’s emphasis on continued or escalated gaming despite negative consequences. These factors highlight the potential dependency on gaming and the manifestation of withdrawal-like symptoms when unable to play. The factors of interpersonal conflicts and social isolation emphasize the impact of problematic gaming on social functioning. These align with the ICD-11’s recognition of gaming disorder as a pattern of behavior where gaming takes precedence over other interests and daily activities, leading to conflicts with the environment and withdrawal from real-life social interactions. In the broader context of the ICD-11, the identified factors of the POGQ provide a nuanced understanding of the dimensions of gaming behavior that may warrant clinical attention. Cognitive-behavioral therapies can be recommended in the management of Problematic online gaming as they allow a focus on these aspects in addition to addressing underlying comorbidities such as depression [79–81]. However, it is important to normalize practices, particularly by utilizing a robust tool like the POGQ. With its construction in six factors, the use of the Arabic version of the POGQ by therapists could focus on a specific problematic dimension and gain a better understanding of the player’s personal gaming experience and reasons for using online video games. The Arabic validation of the POGQ thus enables a more precise detection of problematic use of online video games and the implementation of appropriate treatments to address this issue [82].

It is important to note that like other studies, this one also has limitations. First and foremost, our research design is a cross-sectional, correlational analysis, preventing any inferences about causation. Additionally, respondents participated through online means, raising potential concerns about the authenticity of their identities. Furthermore, the study was conducted exclusively with Moroccan players, necessitating caution in generalizing the results to other cultures. We hope that future studies will replicate these findings in diverse Arab cultures. Another significant consideration is the reliance on self-reported data, highlighting the need for future investigations to validate the identified problematic dimensions through clinical or observational studies. Importantly, our study did not include analyses of convergent, discriminant, or incremental validity. For clinical applications, it is essential that future research incorporates additional analyses, such as assessments of responsivity, sensitivity to change, and the determination of minimally important clinical differences. Exploring these aspects will contribute to a more comprehensive understanding and application of the Arabic version of the POGQ.

## Conclusions

Overall, this work represents the first validation of the Arabic version of the POGQ instrument. We investigated its psychometric properties within a sample of 456 Moroccan university students, employing CFA to investigate its underlying factor structure. The results confirmed its reliability and validity. The Arabic version of POGQ was affirmed to have a six-factor structure, resembling the original POGQ version. These six factors encompassed Withdrawal, Social isolation, Overuse, Immersion, Preoccupation and Interpersonal conflict. Additionally, the Arabic POGQ version demonstrated a strong level of test-retest reliability.

The POGQ scale enables the evaluation of diverse aspects pertaining to online gaming. By utilizing the Arabic version of the POGQ, it becomes feasible to acquire valuable insights into the patterns of gaming addiction and the related interpersonal problems, social isolation, and the degree of withdrawal associated with gaming addiction. The adoption of the POGQ is expected to advance research efforts aimed at addressing and preventing online gaming dependency within the Arabic world.

## Data Availability

The datasets used and/or analyzed during the current study are available from the corresponding author on reasonable request.

## Abbreviations

AVE: Average Variance Extracted
CFA: Confirmatory Factor Analysis
CFI: Comparative Fit Index
CI: Confidence Interval
CR: Composite Reliability
df: Degrees of Freedom
DSM: Diagnostic and Statistical Manual of Mental Disorders
EFA: Exploratory Factor Analysis
GAS: Game Addiction Scale
GFI: Goodness of Fit Index
GSI: Global Severity Index
IC: Interpersonal Conflict
ICC: Interclass Correlation Coefficient
IGD: Internet Gaming Disorder
IM: Immersion
KMO: Kaiser?Meyer?Olkin test
MMORPGs: Massively Multiplayer Online Role Playing Games
NFI: Normed Fit Index
OV: Overuse
PAF: Principal Axis Factoring
PIUQ: Problematic Internet Use Questionnaire
POGQ: Problematic Online Gaming Questionnaire
PR: Preoccupation
RMSEA: Root-Mean Square Error of Approximation
SI: Social isolation
SRMR: Standardized Root-Mean Square Residual
TLI: Tucker Lewis Fit Index
WI: Withdrawal
